# Leveraging Tumor Microenvironment to Boost Synergistic Photodynamic Therapy, Ferroptosis Anti‐Tumor Efficiency Based on a Functional Iridium(III) Complex

**DOI:** 10.1002/advs.202413879

**Published:** 2025-02-14

**Authors:** Yu Pei, Yinzhen Pan, Zhijun Zhang, Jun Zhu, Yan Sun, Qian Zhang, Dongxia Zhu, Guangzhe Li, Martin R. Bryce, Dong Wang, Ben Zhong Tang

**Affiliations:** ^1^ Key Laboratory of Nanobiosensing and Nanobioanalysis at Universities of Jilin Province Department of Chemistry Northeast Normal University 5268 Renmin Street Changchun Jilin Province 130024 P. R. China; ^2^ Center for AIE Research, Guangdong Provincial Key Laboratory of New Energy Materials Service Safety College of Material Science and Engineering Shenzhen University Shenzhen 518060 P. R. China; ^3^ Jilin Provincial Science and Technology Innovation Center of Health Food of Chinese Medicine Changchun University of Chinese Medicine Changchun Jilin Province 130117 P. R. China; ^4^ Department of Chemistry Durham University Durham DH1 3LE UK; ^5^ School of Science and Engineering, Shenzhen Institute of Aggregate Science and Technology The Chinese University of Hong Kong, Shenzhen (CUHK‐Shenzhen) Guangdong 518172 P. R. China

**Keywords:** Fenton reaction, ferrocene, ferroptosis, iridium(III) complex, photodynamic therapy

## Abstract

The tumor microenvironment (TME) severely limits the efficacy of clinical applications of photodynamic therapy (PDT). The development of a functional agent allowing full use of the TME to boost synergistic PDT and ferroptosis anti‐tumor efficiency is an appealing yet significantly challenging task. Herein, to overcome the adverse influence on PDT of hypoxia and high level of glutathione (GSH) in the TME, an imine bond is introduced into an Ir(III)‐ferrocene complex to construct a small molecule drug, named Ir‐Fc, for tumors’ imaging and therapy. The cleavage of the imine bond in the lysosome effectively disrupts the photoinduced electron transfer (PET) process, realizing the decomposition of Ir‐Fc into Fc‐CHO and Ir‐NH_2_. Fc‐CHO produces •OH by Fenton reactions under dark conditions and induces ferroptosis in tumor cells, and Ir‐NH_2_ shows prominent performance for type‐I and type‐II reactive oxygen species (ROS) production. Meanwhile, the ferroptosis pathway simultaneously consumes large amounts of GSH and produces O_2_ for effectively relieving hypoxia. These distinctive outputs make Ir‐Fc an exceptional molecule for effective tumor synergistic therapy. This study thus brings a new and revolutionary PDT protocol for practical cancer treatment.

## Graphical abstract

Ingenious introduction of an imine bond into an Ir(III)‐ferrocene complex effectively disrupts the photoinduced electron transfer process. Allowing full use of the TME to boost the efficacy of PDT, the molecule releases the Ir(III) complex and ferrocene with synergistic enhanced therapeutic effects of PDT and ferroptosis.

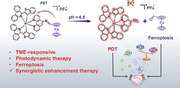

## 1 Introduction

The tumor microenvironment (TME) is considered to be one of the serious obstacles in cancer treatment due to its intrinsic properties of mild acidity, hypoxia, overexpressed glutathione (GSH), and reactive oxygen species (ROS).^[^ ^1^ ^]^ In particular, ROS plays an essential role in each stage of tumor evolution (e.g., tumorigenesis, growth, invasion, and metastasis).^[^ ^2^ ^]^ Nevertheless, once the concentration of ROS in the TME is elevated to an abnormal level, tumor cells can be damaged.^[^ ^3^ ^]^ In this context, ROS‐mediated therapy, so‐called photodynamic therapy (PDT), has shown inexhaustible and vigorous vitality in cancer treatment with the advantages of high selectivity and non‐invasiveness.^[^ ^4^ ^]^ However, the hypoxic characteristic of the TME still limits the efficiency of ROS generation in type‐II PDT, which relies on the presence of molecular oxygen.^[^ ^5^ ^]^ Furthermore, the scavenging of ROS by the overexpressed antioxidant GSH in TME further decreases the efficacy of PDT.^[^ ^6^ ^]^ Hence, it is of fundamental significance to make full use of the unique properties of TME and thus realize efficient PDT. There is particular interest in metal‐based phototherapeutic agents to combat cancer resistance to therapy with current drugs.^[^ ^7^ ^]^

Iron‐dependent ferroptosis is a non‐apoptotic cell death pathway that has attracted much attention since it was first proposed in 2012.^[^ ^8^ ^]^ It can be caused by Fenton reactions, manifested as iron‐dependent accumulation, GSH depletion, and lipid peroxidase (LPO) production, which ultimately leads to a change of mitochondrial morphology.^[^ ^9^ ^]^ Fenton reactions can produce both O_2_ and •OH, which may alleviate the hypoxia of the TME.^[^ ^10^ ^]^ Meanwhile, a large amount of GSH can be consumed in the ferroptosis process, consequently facilitating the accumulation of ROS and enhancing the therapeutic outcomes of PDT.^[^ ^11^ ^]^ However, the •OH produced by Fenton reactions is insufficient to kill the tumor cells.^[^ ^12^ ^]^ The output of •OH from Fenton reactions can be improved by illumination.^[^ ^13^ ^]^ Therefore, the integration of PDT and ferroptosis should be an effective protocol to boost the efficiency of tumor treatment with synergistic rather than merely additive effects. Ji and co‐workers reported a ferritin‐homing nanoparticle (Ce6‐PEG‐HKN_15_) that releases iron that interacts with intracellular H_2_O_2_ to produce O_2_, enhancing PDT and activating endogenous ferroptosis.^[^ ^14^ ^]^ Liu et al. synthesized a type of carrier‐free hybrid nanosphere containing aggregation‐induced emission (AIE) photosensitizers and Fe^3+^, which successively generated O_2_ and depleted overexpressed GSH to modulate TME for efficient PDT.^[^ ^15^ ^]^ Compared with nanomaterials, functional organic or organometallic molecules have well‐defined chemical structures and compositions, so it is relatively easy to study their mechanisms of action and modes of toxicity, which are rarely presented.^[^ ^16^ ^]^

Two major factors affecting the efficiency of ferroptosis are: (i) insufficient endogenous Fe^2+^ in tumor cells;^[^ ^17^ ^]^ (ii) the pH of tumor cells is ≈6–7 which is not sufficiently acidic for the Fenton reactions.^[^ ^18^ ^]^ As a catalyst of Fenton reactions, ferrocene is found to be a potential molecule for inducing ferroptosis.^[^ ^19^ ^]^ Lysosomes, as important organelles at pH 4.5–5.5, play a key role in cell metabolism and death, especially in inducing ferroptosis.^[^ ^20^ ^]^ Ir(III) complexes have been used as potent PSs, not only with high intersystem crossing (ISC) rates but also with the potential to target specific subcellular organelles, which favors biological imaging and cancer therapy.^[^ ^21^ ^]^ Chao et al. reported a mitochondria‐localized Ir(III) complex photosensitizer for two‐photon ferroptosis therapy and photodynamic immunotherapy against melanoma.^[^ ^22^ ^]^ Liu and colleagues developed Ir(III) complex‐based immune agonists that induce ferroptosis through a metal‐ligand synergistic enhancement strategy.^[^ ^23^ ^]^ Structural modification of an Ir(III) complex to acquire multiple cell death pathways is an effective strategy to enhance anti‐tumor efficiency. Ferrocene has been widely used as a building block in medicinal chemistry.^[^ ^24^ ^]^ For example, Sadler et al. recently reported that a conjugated Pt(IV)‐ferrocene molecule induces ferroptosis in vitro upon irradiation.^[^ ^25^ ^]^ Mao's group have reported Ir(III) complexes functionalized with ferrocene to induce immunotherapy/photoimmunotherapy by ferroptosis.^[^ ^16^, ^26^ ^]^ These studies, which involve the ingenious design of molecules with covalent linkage of ferrocene to an Ir(III) complex, demonstrate immunogenic cell death and suppressed tumor growth, and provide enhanced anticancer immunity in vivo by eliciting ferroptosis through a self‐amplifying process. Nevertheless, photoinduced electron transfer (PET) processes that occurred between the ferrocene moiety and the Ir(III) complexes inhibited the PDT process and restricted the synergistic therapeutic effect of PDT and ferroptosis.

**Scheme 1:**
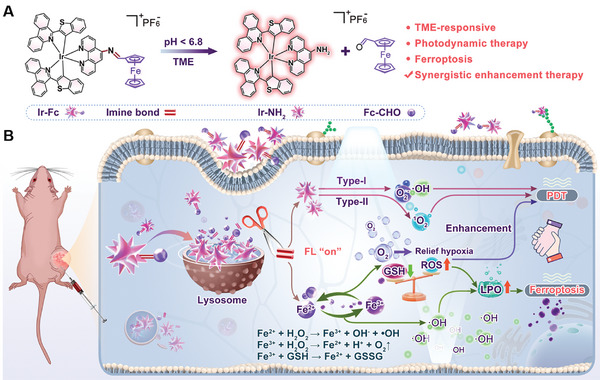
Schematic illustration of the synergistic therapy for tumors. A) Schematic of molecules with pH response. B) Synergistic enhancement of PDT and ferroptosis for treatment in tumors.

Consequently, we envisioned how the characteristics of TME could be utilized to restrict the PET. Our novel molecular design overcomes this major obstacle and leads to unprecedented efficiency of dual‐action PDT and ferroptosis. In contrast to the strong and rigid imidazole or bis(diphenylphosphine) link in the pioneering work of Mao et al.,^[^ ^16^, ^24^ ^]^ we chose an imine link between the ferrocene and Ir(III) complex as it is well known that imine bonds are broken easily under the acidic conditions that are found in lysosomes.^[^ ^27^ ^]^ Herein, an imine bond was used as a linker for complex Ir‐Fc, which was constructed to overcome PET. Complex Ir‐Fc could target lysosomes, and its imine bond was activated by the acidic environment, releasing complex Ir‐NH_2_ and Fc‐CHO (**Scheme** **1**). As the PET process was blocked, Ir‐NH_2_ exhibited enhanced emission and photosensitization properties, which were ideal for tumor imaging and PDT. Complex Ir‐NH_2_ was an excellent PS that generated large amounts of ROS (^1^O_2_, O_2_^•−^, and •OH) through both type‐I and type‐II PDT processes. Meanwhile, Fc‐CHO catalyzed Fenton reactions in lysosomes to generate highly toxic hydroxyl radicals (•OH), which lead to ferroptosis through iron‐dependent accumulation, GSH depletion, and massive production of LPO. Notably, illumination significantly improved the output of •OH. Furthermore, the ferroptosis process relieved the tumor from hypoxia, while GSH depletion facilitated ROS accumulation, both of which enhanced the effects of PDT. Both in vitro and in vivo experiments demonstrated that the complex Ir‐Fc could be successfully used to eliminate 4T1 tumor cells and was non‐toxic to normal cells. This safe and efficient TME‐responsive diagnostic and therapeutic strategy provides innovative insights to achieve highly effective synergistic PDT and ferroptosis.

## 2 Results and Discussion

### 2.1 Molecular Design, Synthesis, and Characterization

**Figure 1:**
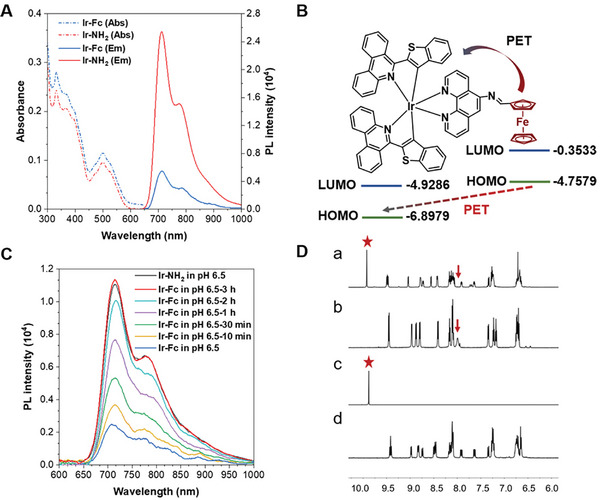
A) Absorption and emission spectra of Ir‐Fc and Ir‐NH_2_ in CH_3_CN solution (10 µM). B) Density functional theory (DFT) calculation shows the mechanisms of the photoinduced electron transfer (PET) in Ir‐Fc. C) Time‐dependent changes in the fluorescence spectrum after the addition of Ir‐Fc (10 µM) in PBS at pH 6.5. D) ^1^H NMR spectra of Ir‐Fc + 0.01 ml HCl (a), Ir‐Fc (b), Fc‐CHO (c) and Ir‐NH_2_ (d). 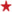: ‐CHO, 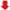: imine bond.

The complex Ir‐Fc was synthesized by a similar method in the literature with only minor modifications (Scheme S1, Supporting Information).^[^ ^28^ ^]^ Ir‐NH_2_ was obtained by adding Ir‐Fc in the acidic buffer solution (pH = 6.5) which cleaved the imine bond. 6‐(Benzo[*b*]thiophen‐2‐yl)phenanthridine (btph) was chosen as a C^N cyclometallating ligand because of its ability to complex with Ir and to induce near infrared (NIR) phosphorescence emission.^[^ ^29^ ^]^ Ir‐Fc and Ir‐NH_2_ were characterized by ^1^H NMR, ^13^C NMR spectroscopy, and mass spectrometry (Figures S1–S8, Supporting Information). The absorption spectra of Ir‐Fc and Ir‐NH_2_ in acetonitrile solution (**Figure** **1**A) display both high‐energy bands (<350 nm) and relatively low‐energy bands (350–600 nm), assigned to the ligand‐centered (^1^LC) π‐π^*^ transitions and mixed metal‐to‐ligand charge transfers (MLCT) and ligand‐to‐ligand charge transfers (LLCT) transitions, respectively.^[^ ^30^ ^]^ The emission of both Ir‐Fc and Ir‐NH_2_ peaks at 712 nm, but the emission intensities show significant differences when excited at 500 nm (Figure 1A; Table S1, Supporting Information).

The weak emission can be attributed to the PET process between the ferrocene moiety and Ir(III) center (Figure 1B). Density functional theory (DFT) calculations also indicate that the PET process occurs between the highest occupied molecular orbital (HOMO) of ferrocene and the HOMO of the Ir(III) complex under photoexcitation, which has the quenching effect on the phosphorescence of Ir‐Fc, resulting in weak emission.^[^ ^16^, ^31^ ^]^ The emission intensity of the complexes gradually increased after incubation in a buffer solution at pH 6.5. As shown in Figure 1C, the complete dissociation of the imine bond in Ir‐Fc required ≈3 h to exhibit full Ir‐NH_2_ emission. This result demonstrated that the slightly acidic environment leads to the successful breaking of the imine bond, turning the pH‐responsive fluorescence emission “on”. Structural changes of Ir‐Fc after acidification were examined using ^1^H NMR. After the addition of the acid, the imine portion corresponding to Ir‐Fc was no longer detected, and the appearance of the aldehyde peak proved the presence of Fc‐CHO after dissociation (Figure 1D). The complete NMR spectra are shown in Figures S9 and S10 (Supporting Information). The results of ^1^H NMR are consistent with emission spectroscopy, validating that Ir‐Fc was successfully activated by acidic pH, potentially overcoming the PET issues discussed above.

### 2.2 Study on PDT Performance of Ir‐Fc

**Figure 2:**
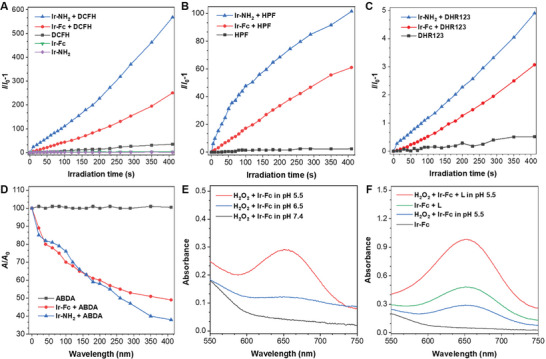
The investigation of ROS generation. A) ROS generation performance of Ir‐NH_2_ and Ir‐Fc (0.1 µm) upon white LEDs (20 mW cm^−2^) with DCFH as indicator. B) •OH generation performance of Ir‐NH_2_ and Ir‐Fc (0.1 µM) upon white LEDs (20 mW cm^−2^) with HPF as indicator. C) O_2_^•ˉ^generation performance of Ir‐NH_2_ and Ir‐Fc (0.1 µM) upon white LEDs (20 mW cm^−2^) with DHR123 as indicator. D) ^1^O_2_ generation performance of Ir‐NH_2_ and Ir‐Fc (0.1 µM) upon white LEDs (20 mW cm^−2^) with ABDA as indicator. E) Absorption spectra of the •OH scavenger 3,3’,5,5’‐tetramethylbenzidine (TMB) after the addition of Ir‐Fc (10 µm) and H_2_O_2_ (90 µM) in PBS at different pH values. F) Absorption spectra of TMB after addition of Ir‐Fc (10 µm) in PBS at pH 5.5, in the presence or absence of H_2_O_2_ (90 µm) or white LEDs (20 mW cm^−2^) (presence = +L).

To verify the efficient PDT potential, the ROS generation efficiency of Ir‐Fc and Ir‐NH_2_ was evaluated by using dichlorodihydrofluorescein diacetate (DCFH‐DA) as an indicator. As shown in **Figures** **2**A and S11 (Supporting Information), the fluorescence intensity of DCFH at 525 nm rapidly increased with illumination in the presence of Ir‐Fc or Ir‐NH_2_, indicating the effective production of ROS. Notably, the ROS generation efficiency of Ir‐NH_2_ was far superior to that of Ir‐Fc, which should be attributed to the fact that the PET process was blocked in the case of Ir‐NH_2_. Then, hydroxyphenyl fluorescein (HPF) (Figure 2B; Figure S12, Supporting Information), dihydrorhodamine 123 (DHR 123) (Figure 2C; Figure S13, Supporting Information), and 9,10‐anthracenediyl‐bis(methylene)‐dimalonic acid (ABDA) (Figure 2D; Figure S14, Supporting Information) were used to check the ROS types. Ir‐Fc and Ir‐NH_2_ generated •OH and O_2_^•¯^ under illumination, with Ir‐NH_2_ showing the superior generation ability. ^1^O_2_ was detected using ABDA as a singlet oxygen scavenger, and significant decreases in the absorbance of ABDA were observed in the presence of the complex under illumination, indicating the production of ^1^O_2_. Meanwhile, Ir‐NH_2_ exhibits good photostability under the illumination, which is one of the most important criteria for PSs (Figure S15, Supporting Information). These results confirm that Ir‐NH_2_ could be applied to PDT as an excellent photosensitizer with type‐I and type‐II ROS simultaneously.

### 2.3 Fenton Catalytic Activity of Ir‐Fc

Since the ferrocene should confer the Fenton catalytic properties to Ir‐Fc, we next investigated the ability of Ir‐Fc to produce •OH under different conditions through 3,3′,5,5′‐tetramethylbenzidine (TMB) experiments. TMB is a widespread indicator for detecting •OH, and its solution shows a recognizable absorption band at 650 nm when oxidized.^[^ ^32^ ^]^ The essential requirements for Fenton reactions are Fe^2+^, H_2_O_2,_ and an acidic environment (Figure S16, Supporting Information). As illustrated in Figure 2E, Ir‐Fc could efficiently react with H_2_O_2_ to generate •OH at pH 5.5. In contrast, Ir‐Fc did not display catalytic activity at pH 7.4 and generated only limited amount of •OH at pH 6.5. The higher •OH‐generating capacity of Ir‐Fc was detected at pH 5.5, which is close to the lysosomal pH (≈pH 4.5–5.5). Due to the type‐I PDT process of Ir‐Fc under illumination, the TMB experiments also verified the production of •OH (Figure 2F). Moreover, when Ir‐Fc was exposed to illumination under Fenton‐catalyzed conditions, the absorption intensity of TMB was abruptly enhanced, suggesting that illumination promoted the production of •OH in the Fenton reactions (Figure 2F).

### 2.4 Localization in Lysosomes

**Figure 3:**
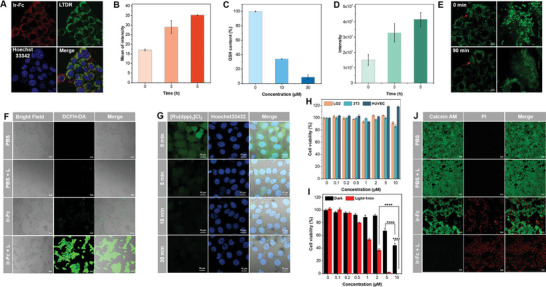
In vitro ferroptosis and PDT cellular tumoricidal performance evaluation. A) Colocalization images of Ir‐Fc (30 µm, 1 h) with LysoTracker Deep Red (LTDR) in 4T1 cells. B) Intracellular Fe^2+^ production at different incubation times (mean ± SD, *n* = 3). C) Intracellular GSH depletion with different concentrations of Ir‐Fc (0–30 µM) (mean ± SD, *n* = 3). D) Impact of Ir‐Fc (30 µM) on the level of cellular LPO (mean ± SD, *n* = 3). E) Real‐time tracking of mitochondrial morphology in 4T1 cells treated with Ir‐Fc (10 µM) for 90 min. F) Fluorescence microscope images of 4T1 cells incubated with DCFH‐DA in different groups (PBS, PBS + L, Ir‐Fc, Ir‐Fc + L), both with or without white LEDs (20 mW cm^−2^). Scale bars = 50 µm. G) 4T1 cells were stained with [Ru(dpp)_3_]Cl_2_ as dissolved O_2_ probe and then treated with Ir‐Fc for different incubating times (0, 5, 15, and 30 min). Scale bars = 10 µm. H) Cell viability of LO2, 3T3, and HUVEC cells treated with different concentrations of Ir‐Fc (mean ± SD, *n* = 6). I) Cell viability of 4T1 cells treated with different concentrations of Ir‐Fc in the absence and presence of white LEDs (20 mW cm^−2^) (^****^ *p * < 0.0001) (mean ± SD, *n* = 6). J) Live/dead cell staining of 4T1 cells treated with PBS and Ir‐Fc in the absence and presence of white LEDs (20 mW cm^−2^), with FDA/PI double‐staining method. Scale bar: 50 µm.

To investigate the anti‐tumor mechanism of Ir‐Fc, its intracellular localization was first studied by commercial organelle‐specific staining probes. After incubation with 4T1 cells for 1 h, the overlap of red fluorescence and green fluorescence indicated that Ir‐Fc was mainly localized in lysosomes (**Figure** **3**A). Then, with the addition of the acidic inhibitor Bafilomycin A1 (BafA1), the fluorescence of Ir‐Fc and the fluorescence of LysoTracker Deep Red were significantly decreased (Figure S17, Supporting Information). These results indicated that because lysosomal acidity was inhibited by BafA1, the imine bond of Ir‐Fc was not broken, and thus fluorescence‐enhancing Ir‐NH_2_ was not released. It is further demonstrated that the low pH of the acidic organelle lysosome promoted rapid dissociation of Ir‐Fc and subsequent Fenton reactions.

### 2.5 Efficacy of Ir‐Fc‐Induced Ferroptosis

Ferroptosis is an iron‐dependent form of cell death in which Fe^2+^ plays a vital role.^[^ ^33^ ^]^ In the preliminary step, FerroOrange was utilized as a fluorescent probe to measure intracellular Fe^2+^ levels. It was visualized that orange fluorescence gradually increased in 4T1 cells after treatment with Ir‐Fc, which confirmed the presence of intracellular Fe^2+^ and laid the foundation for the Fenton reactions (Figure 3B; Figure S18, Supporting Information). Next, to observe the production of intracellular ROS during PDT and Fenton reactions, DCFH‐DA was selected as the fluorescent probe. As shown in Figure 3F, green fluorescence was observed in Ir‐Fc‐incubated 4T1 cells under dark conditions, suggesting the production of •OH by Fe^2+^‐mediated Fenton reactions.

GSH is an antioxidant in tumor cells that scavenges ROS generated during PDT. The depletion of GSH is considered an essential marker of ferroptosis.^[^ ^34^ ^]^ The GSH‐depleting activity of Ir‐Fc in 4T1 cells was verified by intracellular GSH content assay. In dark conditions, as the concentration of Ir‐Fc increased, intracellular GSH levels decreased substantially (Figure 3C) which will both facilitate the accumulation of ROS during PDT, and also further promote the accumulation of LPO to induce ferroptosis. Liperfluo, which is oxidized by LPO to produce green fluorescence was used as a probe to assess intracellular LPO accumulation. The intensity of green fluorescence in 4T1 cells increased markedly with the extended incubation time of Ir‐Fc in the dark (Figure S19, Supporting Information). Figure 3D showed a histogram of the relative intensity of LPO, indicating a significant accumulation of LPO within the cell. Mitochondrial dysfunction is one of the key indicators to validate ferroptosis.^[^ ^35^ ^]^ Thus, the mitochondrial membrane potential (MMP) was detected using JC‐1 as a fluorescent probe that shows red fluorescence in intact mitochondria, and green fluorescence in depolarized mitochondria. As shown in Figure S20 (Supporting Information), obvious MMP loss was observed in Ir‐Fc‐treated cells under dark conditions. Green fluorescence increased markedly after imposed illumination, indicating that illumination promoted ferroptosis. Finally, the alternations in mitochondrial morphology upon Ir‐Fc treatment were detected by confocal microscopic imaging. Compared with the control group, the morphology of mitochondria changed from reticular structure to a punctate structure, indicating that the mitochondria were damaged and disrupted (Figure 3E; Figure S21, Supporting Information). This is consistent with the morphological changes in mitochondria induced by ferroptosis reported in the literature.^[^ ^26^ ^]^

### 2.6 Efficacy of Ir‐Fc‐Induced PDT

The intracellular O_2_ generation capacity of Ir‐Fc was assayed by the hypoxia probe [Ru(dpp)_3_]Cl_2_. As shown in Figure 3G, [Ru(dpp)_3_]Cl_2_ emitted strong green fluorescence under hypoxic conditions. The green fluorescence in Ir‐Fc‐treated 4T1 cells under dark conditions decreased rapidly with time, suggesting that Ir‐Fc produced O_2_ through the Fenton reactions and alleviated hypoxia of tumor cells. Thus, Ir‐Fc successfully induced intracellular O_2_ production, which effectively enhanced type‐II PDT. As in **Figure 3F,** strong ROS generation was detected upon illumination, which could be attributed to powerful PDT effects and also implies the synergistic enhancement effect of PDT and ferroptosis.

**Figure 4:**
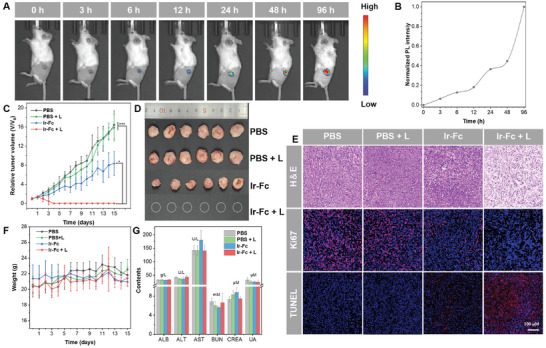
In vivo imaging and antitumor evaluation of Ir‐Fc on 4T1‐tumor‐bearing mice. A) In vivo fluorescence imaging at various time points post‐injection of Ir‐Fc. B) PL intensity at different times following injection of Ir‐Fc. C) Time‐dependent tumor growth curves of 4T1 tumor‐bearing mice with various treatments (^*^ *p* < 0.05, ^***^ *p* < 0.001) (mean ± SD, *n* = 6). D) Tumor images of 4T1 tumor‐bearing mice subjected to various treatments. E) H&E, TUNEL, and Ki67 staining analyses of tumor tissues following diverse therapeutic interventions. F) Body weights of mice in each treatment group (mean ± SD, *n* = 6). G) Biochemical parameters of hepatic function markers and renal function markers of 4T1 tumor‐bearing mice on day 15 post different interventions (mean ± SD, *n* = 6).

### 2.7 Cytotoxicity of Ir‐Fc

Inspired by the excellent PDT and ferroptosis properties of Ir‐Fc, synergistic therapeutic effects were evaluated in tumor cells. First, Ir‐Fc showed no significant toxicity to normal cells (LO2, 3T3, and HUVEC) under dark conditions, proving its excellent biocompatibility and biosafety (Figure 3H). Cell viability was determined by CCK‐8 assay in 4T1 cells following treatment with Ir‐Fc in the absence or presence of illumination. As shown in Figure 3I, compared with the control group, Ir‐Fc‐treated dark groups showed significant cytotoxicity, with cell viability reduced to less than 50%, indicating that Ir‐Fc inhibited tumor cell growth through ferroptosis under dark conditions. Interestingly, following the illumination treatment, the viability of 4T1 cells decreased amazingly to less than 1%, demonstrating not only the high phototoxicity of Ir‐Fc, but also the excellent synergistic toxicity of PDT and ferroptosis. Therefore, we infer that Fenton reactions promote O_2_ generation, and ferroptosis depletes GSH, both of which enhance the generation and accumulation of ROS for the PDT process. At the same time, illumination also promotes the generation of •OH in the Fenton reactions, enhancing the efficiency of ferroptosis, and achieving ultra‐high tumoricidal efficacy on 4T1 cells through synergistic treatment. To demonstrate the synergistic effect of Ir‐Fc, we assessed cytotoxicity using a fluorescein diacetate/propidium iodide (FDA/PI) double‐staining method to observe live (green) and dead (red) 4T1 cells. Figure 3J shows that cells treated with Ir‐Fc exhibited significantly increased red fluorescence under dark conditions, and about half of the cells were killed. Strikingly, almost all 4T1 cells incubated with Ir‐Fc followed by illumination were killed, consistent with the cytotoxicity results. The results indicate that the combination of PDT and ferroptosis enhances the cytotoxicity in 4T1 tumor cells.

### 2.8 In Vivo Synergistic Therapeutic Effects

The ultimate goal of developing therapeutic agents is to improve tumoricidal effects in vivo. Initially, the ability to image fluorescence in vivo was investigated by intratumorally injecting Ir‐Fc into tumor‐bearing mice. As shown in Figure 4A, a clear fluorescence signal was observed at 6 h postinjection, indicating that Ir‐Fc gradually dissociated in the acidic environment of the tumor to release Ir‐NH_2_ with enhanced emission. The fluorescence signal in the tumor region was gradually enhanced with time, reaching a maximum at 96 h after injection (Figure 4B). After confirming the imaging ability of Ir‐Fc, the synergistic anticancer effect was further explored by using BALB/c nude mice bearing 4T1 xenograft tumors. Mice were randomly divided into 4 groups (n = 6 mice per group). The group injected with PBS was defined as a negative control, and tumor volumes were recorded and shown in Figure 4C. Compared with the control group, the Ir‐Fc group (without illumination) showed a tumor suppression effect, which was attributed to the ferroptosis triggered by the ferrocene‐induced Fenton reactions under dark conditions. In particular, almost all solid tumors were eliminated in Ir‐Fc plus illumination‐treated mice, and scarcely any tumors were observed at day 4 of treatment. As expected, treatment with Ir‐Fc plus illumination resulted in the highest rate of tumor inhibition, which was consistent with the in vitro results. The complete disappearance of the tumor without recurrence within 15 days strongly demonstrated the excellent synergistic PDT‐ferroptosis therapeutic efficiency of Ir‐Fc in vivo (Figure 4D). Afterward, histological and immunohistochemical analyses further verified the synergistic mechanism of Ir‐Fc in vivo. Hematoxylin and eosin (H&E) staining experiments showed some void spaces in the cells of the Ir‐Fc group compared to the tightly arranged tumor cells in the control group, suggesting that the tumor tissues were partially disrupted under dark conditions (Figure 4E). Furthermore, significant abnormalities could be observed after Ir‐Fc plus illumination, and most of the tumor cells underwent severe damage, indicating the dual action of PDT and ferroptosis. Concurrently, a remarkable trend of apoptosis and suppressed cell proliferation of tumors in the presence of illumination could also be observed in TUNEL and Ki67 staining analyses (Figure 4E). All the above results support the exciting synergistic therapeutic effects of Ir‐Fc.

### 2.9 Biosafety Analysis

The biological safety of therapeutic agents is important, so the potential toxicity of Ir‐Fc in vivo was systematically evaluated. During the treatment period, there was no noticeable change in the body weight of mice in each group, which implies that Ir‐Fc is safe in vivo (Figure 4F). At the end of the 15‐day treatment, mice were sacrificed, and subjected to hematological and biochemical analyses. There were no statistical differences in the detailed biochemical parameters of hepatic and renal function markers [including albumin (ALB), alanine aminotransferase (ALT), aspartate aminotransferase (AST) and urea nitrogen (BUN), creatinine (CREA) and uric acid (UA)] in the Ir‐Fc alone administered group and the Ir‐Fc plus illumination group, compared to the control group: these results demonstrate the absence of significant hepatotoxicity and nephrotoxicity (Figure 4G). In routine blood examination, those indexes in the Ir‐Fc plus illumination group were in the normal range. However, the test indexes in the Ir‐Fc darkness group showed abnormalities, possibly due to the inefficiency of the single‐modality treatment (Table S2, Supporting Information). It was further evidence of the efficiency and safety of synergistic therapy. The results of histological section analysis are additional evidence of the low toxicity of Ir‐Fc. H&E staining of vital organs revealed no noticeable inflammatory lesions or damage to the heart, liver, spleen, lung, or kidney (Figure S22, Supporting Information). The remarkable biosafety of Ir‐Fc is crucial for its synergistic therapeutic applications.

## 3 Conclusion

In summary, we have fully utilized the characteristics of the TME to design and synthesize a pH‐responsive Ir‐Fc complex. Ir‐Fc dissociates within the lysosome, thus overcoming the PET process and enhancing the emission and photosensitizing properties. In the presence of illumination, Ir‐Fc generated large amounts of ROS by type‐I and type‐II PDT processes. Under dark conditions, Fe^2+^ in Ir‐Fc catalyzed Fenton reactions to generate Fe^3+^ and toxic •OH, and Fe^3+^ was further converted to Fe^2+^ via GSH‐mediated redox reactions. The reaction pathways consumed high amounts of GSH and promoted the continued generation of •OH by Fenton reactions, leading to the accumulation of LPO, which triggered ferroptosis. Meanwhile, Fenton reactions generated O_2_ to overcome the tumor hypoxic microenvironment. The depletion of GSH and generation of O_2_ both contributed to the efficiency of PDT, realizing the targeted synergistic enhancement effect of PDT and ferroptosis. Ir‐Fc achieved significant tumor suppression and ablation both in vivo and in vitro with excellent biocompatibility. Ir‐Fc represents a novel agent that integrates tumor diagnosis and treatment with many exceptional features such as lysosomal targeting, acidic pH response, and synergistic enhancement of treatment, illustrating a promising and versatile strategy to achieve outstanding anti‐tumor effects.

## Conflict of Interest

The authors declare no conflict of interest.

## Supporting information



Supporting Information

## Data Availability

The data that support the findings of this study are available in the supplementary material of this article.
