# Supplementary material for: Leveraging Tumor Microenvironment to Boost Synergistic Photodynamic Therapy, Ferroptosis Anti‐Tumor Efficiency Based on a Functional Iridium(III) Complex
Source: Adv Sci (Weinh). 2025 Feb 14;12(14):2413879. doi: 10.1002/advs.202413879 (PMC11984874; doi:10.1002/advs.202413879)
Supplement: Supplementary file 1 — Supporting Information [file ADVS-12-2413879-s001.pdf]

ADVANCED SCIENCE
Open Access

Supporting Information

for *Adv. Sci.*, DOI 10.1002/advs.202413879

Leveraging Tumor Microenvironment to Boost Synergistic Photodynamic Therapy, Ferroptosis Anti-Tumor Efficiency Based on a Functional Iridium(III) Complex

Yu Pei, Yinzhen Pan, Zhijun Zhang, Jun Zhu, Yan Sun, Qian Zhang, Dongxia Zhu, Guangzhe Li*, Martin R. Bryce*, Dong Wang* and Ben Zhong Tang**

Supporting Information
©Wiley-VCH 2024
69451 Weinheim, Germany

Leveraging Tumor Microenvironment to Boost Synergistic Photodynamic Therapy and Ferroptosis Anti-Tumor Efficiency Based on a Functional Iridium(III) Complex

Abstract: The tumor microenvironment (TME) severely limits the efficacy of clinical applications of photodynamic therapy (PDT). The development of a functional agent allowing full use of the TME to boost synergistic PDT and ferroptosis anti-tumor efficiency is an appealing yet significantly challenging task. Herein, to overcome the adverse influence on PDT of hypoxia and high level of glutathione (GSH) in the TME, we introduced an imine bond into an Ir(III)-ferrocene complex to construct a small molecule drug, named Ir-Fc, for tumors' imaging and therapy. The cleavage of the imine bond in the lysosome effectively disrupts the photoinduced electron transfer (PET) process, realizing the decomposition of Ir-Fc into Fc-CHO and Ir-NH_2. Fc-CHO produces •OH by Fenton reactions under dark conditions and induces ferroptosis in tumor cells, and Ir-NH_2 shows prominent performance for type-I and type-II reactive oxygen species (ROS) production. Meanwhile, the ferroptosis pathway simultaneously consumes large amounts of GSH and produces O_2 for effectively relieving hypoxia. These distinctive outputs make Ir-Fc an exceptional molecule for effective tumor synergistic therapy. This study thus brings a new and revolutionary PDT protocol for practical cancer treatment.

Table of Contents

Experimental Procedures S2
Synthesis and Characterization S3
Scheme S1. The synthetic routes S4
Figure S2-S8. NMR and HRMS spectra of intermediates and products S5
Figure S9. ROS generation measurement S9
Figure S10. Hydroxyl radical generation measurement S9
Figure S11. Superoxide anion generation measurement S9
Figure S12. Singlet oxygen generation measurement S9
Figure S13. Absorption spectra of TMB solutions at different pH values S10
Figure S14. Lysosomal targeting analysis in cells S10
Figure S15. Confocal images of intracellular Fe^{2+} S10
Figure S16. Confocal images of lipid peroxides S11
Figure S17. Mitochondrial morphology in cells S12
Figure S18. Histological images of organs in 4T1 tumor-bearing mice S13
Table S1. Photophysical characteristics S13
Table S2. Routine blood indexes of mice after different treatments. S14
References S14

Experimental Procedures

Main materials

Commercially available chemicals, such as 1,10-phenanthrolin-5-amine, ferrocenecarboxaldehyde, benzo[*b*]thiophen-2-ylboronic acid, 6-chlorophenanthridine, $IrCl_3$•H_2O *et al*. were obtained from Adamas, Energy, TCI and Bide and used as received unless otherwise stated. Phosphate buffered saline (PBS, pH 7.4), 2',7'-dichlorodihydrofluorescein diacetate (DCFH-DA), fluorescein diacetate (FDA), propidium iodide (PI), 3'-(*p*-hydroxyphenyl) fluorescein (HPF), 9,10-anthracenediyl-bis(methylene) dimalonic acid (ABDA) were purchased from Sigma-Aldrich. LysoTracker Green was purchased from Thermo Fisher Scientific. Hoechst 33342 were purchased from Dojindo Laboratories. FerroOrange was bought from Dojindo. $[Ru(dpp)_3]Cl_2$ was obtained from Bestbio. Roswell Park Memorial Institute (RPMI-1640) medium, Dulbecco's Modified Eagle's Medium (DMEM) medium, fetal bovine serum (FBS), penicillin and streptomycin were purchased from Gibco. Reactive oxygen species (ROS) detection kit was purchased from Beyotime.

Instruments

Reactions were monitored with analytical thin-layer chromatography (TLC) on silica. ^{1}H NMR and ^{13}C NMR data were recorded on Bruker nuclear magnetic resonance (500 MHz and 600 MHz) spectrometers unless otherwise specified, respectively. High-resolution mass spectra (HRMS) were recorded on a GCT premier CAB048 mass spectrometer operating in a MALDI-TOF mode. Absorption spectra were measured on a PerkinElmer Lambda 950 spectrophotometer or a Milton Roy Spectronic 3000 array spectrophotometer. Steady-state fluorescence spectra were recorded on a Perkin-Elmer LS 55 spectrofluorometer. The absolute PLQY was determined by a Hamamatsu quantum yield spectrometer C11347 Quantaurus QY. Photodynamic experiments were implemented by using white LEDs. The cell viability was detected by CCK-8 kit, and the absorbance of each sample was measured at 450 nm using a microplate reader (BioTek). The cellular fluorescence images were taken by confocal laser scanning microscope (CLSM, ZEISS-LSM880).

DFT calculations

The ground state and excited state optimizations of Ir-Fc used DFT methods in the Gaussian09 program with SMD solvation model (dichloromethane). The 6-31G** basis set was used for the C, H, O, N, S atoms, and the Stuttgart−Dresden double-zeta (SDD) basis set5 with an effective core potential (ECP) was used for the Ir and Fe atoms.

Detection of ROS generation

The ROS generation efficiency in solution was investigated by using the common DCFH-DA as indicator. Briefly, 0.5 mL DCFH-DA in ethanol (1×10^{-3} M) was added to 2 mL 1×10^{-2} M NaOH and stirred at room temperature for 30 min. By that time, DCFH-DA was hydrolyzed to DCFH. Then the hydrolysate was neutralized with 10 mL of PBS at pH 7.4, and kept in the dark until use. The activated DCFH-DA solution (DCFH, 5×10^{-6} M) was added into the sample solution containing Ir-Fc or Ir-NH_2 (1×10^{-7} M). Afterward, the mixed solution was treated with white LED irradiation for different time intervals. The fluorescence of the indicator at 525 nm triggered by complex-sensitized ROS was measured in a PL instrument at the excitation wavelength of 488 nm.

To further distinguish the type of ROS generated by Ir-Fc and Ir-NH_2, ABDA, DHR123 and HPF were used as the other three indicators to evaluate the singlet oxygen, superoxide anion and hydroxyl radical generation ability of the Ir-Fc and Ir-NH_2 under white LED irradiation, respectively. In brief, ABDA (5×10^{-6} M) solution was mixed with the Ir-Fc and Ir-NH_2 in water (1×10^{-7} M) and then exposed to white LEDs. The absorbance decrease of ABDA at 378 nm was recorded at various irradiation times. Ir-Fc and Ir-NH_2 (1×10^{-7} M) were added to PBS buffer solution of DHR123 for the same illumination conditions. PBS buffer solution containing HPF (5×10^{-6} M) was added with Ir-Fc and Ir-NH_2 (1×10^{-7} M) and then subjected to the same illumination conditions. The fluorescence signal of the indicator was monitored in a range of 500-600 nm with the excitation wavelength at 490 nm. The fluorescence intensity at 515 nm was recorded to indicate the superoxide anion and hydroxyl radical generation rates.

Catalyzing of Fenton reactions

Ir-Fc (10 μM), TMB (20 μM), and H_2O_2 (90 μM) were mixed in buffer solutions (pH 5.5, 6.5, and 7.4) in the dark and the absorption peaks were tested for changes at 650 nm using UV-Vis absorption spectroscopy.

Intracellular tracking

Mouse breast cancer 4T1 cell line was purchased from Chinese Academy of Science Cell Bank for Type Culture Collection, and grown in 1640 culture medium containing 10% FBS and 1% antibiotics (penicillin-streptomycin) at 37 °C in a humidified environment of 5% CO_2. 4T1 cells were seeded at a suitable density in a glass bottom dish and cultured for 24 h. The cells were then incubated with fresh medium containing Ir-Fc (30 μM) for 3 h and washed with PBS, followed with LysoTracker Green (LTG) and Hoechst 33342 for 30 min. After that, the samples were washed with PBS and imaged by CLSM to investigate the subcellular localization of TSSI NPs. Conditions: excitation wavelength: 405 nm for Hoechst 33342, 488 nm for LysoTracker Green, and 633 nm for TSSI NPs; emission filter: 410-500 nm for Hoechst 33342, 500-550 nm for LysoTracker Green, and 650-750 nm for TSSI NPs.

Fe^{2+} content assay

FerroOrange was used as the probe to test the Fe^{2+} content in cells. Briefly, 4T1 cells were seeded in 2.0 cm-confocal dishes (2.0×10^5 per dish) and incubated for 24 h. Then, the cells were incubated with Ir-Fc (30 μM). The cells were washed with HBSS, and they were stained with FerroOrange at 37 °C for 30 min. Finally, the cells were imaged by using CLSM.

GSH content assay

4T1 cells were seeded in 6 cm dishes. When the density reached about 80%, the cells were treated with Ir-Fc at different concentrations (0 μM, 10 μM and 30 μM). The GSH Assay Kit (Beyotime, China) following the manual protocols.

Lipoperoxide content assay

4T1 cells were seeded into the cell culture dishes treated with Ir-Fc (30 μM) to detect the existence of lipoperoxide. When the cell density reached about 65% after 24 h of different treatments, 4T1 cells were incubated with lipoperoxide indicator Liperfluo (10 μM) in the culture medium at 37 °C, followed by CLSM analysis.

Intracellular ROS generation

4T1 cells were seeded and cultured in a glass bottom dish for 24 h. Then, the cells were treated with Ir-Fc (30 μM) in fresh medium for 12 h. Afterward, the cells were incubated with 1 mL fresh serum-free medium containing 10 μM DCFH-DA at 37 °C for 20 min. After washing, the cells were incubated in dark conditions for 0.5 h at 37 °C for CLSM imaging. Then, the cells were illuminated using white LEDs for 10 min and then incubated at 37 °C for another 0.5 h before CLSM imaging. Conditions: excitation wavelength 488 nm, emission filter 500-550 nm.

Detection of O_2 production

The O_2 generation ability of Ir-Fc *in vitro* can be detected using $[Ru(dpp)_3]Cl_2$. The HeLa cells were incubated in 6-well plates for 12 h and then 100 μL of $[Ru(dpp)_3]Cl_2$ (50 μM) PBS solution was added into the cells and incubation continued for another 4 h. Then, 0.6 mL PBS solution containing 30 μM of Ir-Fc was added into the each well and incubated for different times (0, 5, 15, and 30 min). Finally, the cells were stained with Hoechst 33432 for 15 min and observed by CLSM.

Live-dead cell staining

The 4T1 cells were seeded and cultured in a glass bottom dish for 24 h, Ir-Fc (30 μM) was then added into the cell culture medium. After 12 h incubation, the cells were washed and replaced with fresh medium, then incubated for 1 h at 37 °C under dark conditions. After that, the cells were incubated at 37 °C for another 1 h, then successively stained with PI (60 μg/mL) and FDA (100 μg/mL) in PBS for 10 min. Subsequently, the cells were gently washed with PBS and then imaged by CLSM. Afterwards, the above protocol was repeated after illumination with white LEDs for 1 min and imaged with CLSM. Conditions: excitation wavelength: 488 nm for FDA and 534 nm for PI; emission filter: 500-550 nm for FDA and 550-650 nm for PI.

Biocompatibility evaluation

LO2, 3T3 and HUVEC cells were purchased from Chinese Academy of Science Cell Bank for Type Culture Collection and grown in DMEM culture medium containing 10% FBS and 1% antibiotics (penicillin-streptomycin) at 37 °C in a humidified environment of 5% CO_2. Then, the cells in a logarithmic growth phase were harvested and seeded in 96-well plates at a density of 5×10^3 cells/well for 24 h incubation. Subsequently, the medium was replaced with the fresh medium containing different concentrations of Ir-Fc. After further incubation for 24 h, the medium was removed and washed with PBS three times. Cells were then incubated with fresh serum-free medium containing 10% CCK-8 for 2 h in the dark. Finally, the absorbance of the products was measured at a wavelength of 450 nm by a microplate reader. The results were expressed as the viable percentage of cells after different treatments relative to the control cells without any treatment. The relative cell viability was calculated according to the following formula: Cell viability (%) = (ODsample - ODbackground)/(ODcontrol - Dbackground) × 100%.

Cytotoxicity test

4T1 cells were seeded in 96-well plates at a density of 5×10^3 cells/well and incubated for 24 h. Then the cells were incubated with different concentrations of Ir-Fc in fresh medium. After 12 h incubation, the cells were exposed to white LEDs for 5 min. After further incubation for 12 h, the medium was removed and washed with PBS three times. The following steps for CCK-8 test were carried out as in the above-mentioned procedures.

Animals and tumor models

BALB/c nude mice (~4 weeks old) were purchased from Beijing Vital River Laboratory Animal Technology. All animals were acclimatized to the animal facility for one week prior to experimentation and housed under pathogen-free conditions. All animals were fed under conditions of 25 °C and 55 % humidity and allowed free access to standard laboratory water and food. All the animals' operation complied with the regulations of the Animal Ethical and Welfare Committee of Shenzhen University (AEWC-SZU). The xenograft tumor models were established by subcutaneous injection of 5×10^5 4T1 cells in PBS buffer into the right flanks of each mouse. After about 10 days, mice with tumor volumes at about 100 mm^3 were used subsequently.

***In vivo* fluorescence imaging**

The 4T1 tumor-bearing mice were administered Ir-Fc-containing saline by intratumoral injection into each mouse. Then, at 3, 6, 12, 24, 48 and 96 h post-injection, the mice were anesthetized using 2 % isoflurane in oxygen and imaged through a commercial imaging system.

***In vivo* antitumor efficacy**

When the inoculated tumor volumes reached about 100 mm^3, 24 mice were randomly divided into 4 groups each of 6 mice, named "PBS", "PBS + L", "Ir-Fc" and "Ir-Fc + L", respectively. On day 0, for "PBS" and "Ir-Fc" groups, 200 μL of PBS and Ir-Fc (1 mM) were separately injected into the 4T1 tumor-bearing mice without subsequent irradiation. In case of "PBS + L" and "Ir-Fc + L" groups, after injection of PBS and Ir-Fc (1 mM) for 24 h, respectively, the tumors of mice in each group were continuously irradiated with white LEDs for 15 min. After a variety of treatments, the mouse body weight and tumor volume were recorded every 3 days during 15-days study duration. The tumor volume was measured by a vernier caliper and calculated as $V = a \times b^2/2$. (a: tumor length; b: tumor width). The relative tumor volume was calculated as $RTV = (V-V_0)/V_0$ (V_0 was the initial tumor volume). The relative body weight was calculated as $RBW = (W-W_0)/W_0$ (W_0 was the initial mouse body weight).

Histological and hematological analyses

At day 15 of various treatments, the blood samples of the mice were collected into blood collection tubes for serum biochemistry and hematology analyses. Then, all the tumors as well as major organs (heart, liver, spleen, lung and kidney) of the mice in each group were collected, fixed in 4 % (v/v) formalin saline overnight, embedded in paraffin and then sectioned at the thickness of 5 μm. For the "Ir-Fc + L" group, tumor samples were obtained at 12 h after irradiation. Subsequently, the obtained tumor sections of these four groups were subjected to H&E, Ki67 and TUNEL staining and then examined with an inverted optical microscope for histopathological evaluation. Additionally, the slices of major organs were administrated with H&E staining for biosafety evaluation.

Statistical Analysis

Data are denoted as the mean ± standard deviation (SD). The significance between experimental and control groups was determined by unpaired 2-tailed Student's t-test using the GraphPad Prism 7. A value of $p < 0.05$ was considered statistically significant. *$p < 0.05$, **$p < 0.01$, ***$p < 0.001$, ****$p < 0.0001$.

Synthesis and characterization

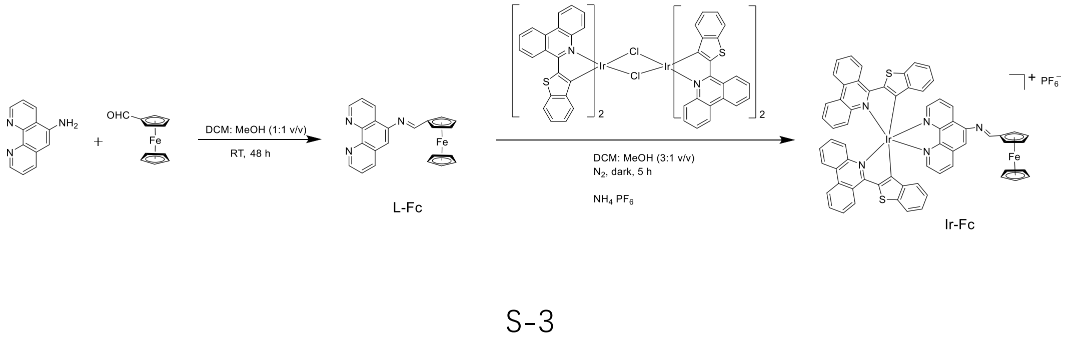

SUPPORTING INFORMATION

Scheme S1. The synthetic route to Ir-Fc.

Synthesis of L-Fc: 1,10-Phenanthrolin-5-amine (1.95 g, 10 mmol) and ferrocenecarboxaldehyde (2.14 g, 10 mmol) were added into a 100 mL two-necked round-bottom flask, and the flask was vacuumed and purged with dry nitrogen three times. Then CH_2Cl_2 (15 mL) and MeOH (15 mL) were added, and the mixture was stirred for 48 h at room temperature. At the end of the reaction, the solvent was evaporated under reduced pressure. The crude product was purified by column chromatography on silica gel to afford product as a red solid (54% yield). **^{1}H NMR (600 MHz, DMSO-*d*$_6$)** δ 9.14 (dd, *J* = 4.2, 1.7 Hz, 1H), 9.01 (dd, *J* = 4.2, 1.6 Hz, 1H), 8.69 (d, *J* = 10.3 Hz, 2H), 8.51 (dd, *J* = 8.0, 1.3 Hz, 1H), 7.81 (dd, *J* = 8.2, 4.2 Hz, 1H), 7.74 (dd, *J* = 8.0, 4.3 Hz, 1H), 7.56 (s, 1H), 4.96 (t, *J* = 1.7 Hz, 2H), 4.64 – 4.62 (m, 2H), 4.35 (s, 5H).

Synthesis of $Ir_2(btph)_4\mu$-Cl_2: The chloro-bridged precursor $Ir_2(btph)_4\mu$-Cl_2 was synthesized by literature methods.[1] **^{1}H NMR (500 MHz, $CDCl_3$)** δ 8.94 (d, *J* = 7.9 Hz, 1H), 8.42 (d, *J* = 8.2 Hz, 1H), 7.97 (d, *J* = 8.6 Hz, 2H), 7.90 (t, *J* = 7.8 Hz, 1H), 7.79 (t, *J* = 7.4 Hz, 1H), 7.42 (d, *J* = 7.9 Hz, 1H), 6.71 (t, *J* = 7.4 Hz, 1H), 6.58 (t, *J* = 7.5 Hz, 1H), 5.97 (t, *J* = 7.6 Hz, 1H), 5.80 (t, *J* = 8.2 Hz, 1H), 5.39 (d, *J* = 8.2 Hz, 1H).

Synthesis of Ir-Fc: $Ir_2(btph)_4\mu$-Cl_2 (215.2 mg, 0.2 mmol) and L-Fc (195.5 mg, 0.5 mmol) were refluxed in a mixed solution (12 mL) containing CH_2Cl_2 and MeOH (v/v = 3/1) for 5 h protected from light. After cooling to room temperature, NH_4PF_6 (10 eq) was added and stirring continued for 0.5 h. The crude product was purified by column chromatography on silica gel to afford product as a red solid (39% yield). **^{1}H NMR (600 MHz, DMSO-*d*$_6$)** δ 9.45 (d, *J* = 8.2 Hz, 2H), 8.97 (d, *J* = 4.7 Hz, 2H), 8.88 (d, *J* = 8.2 Hz, 2H), 8.80 (d, *J* = 8.3 Hz, 2H), 8.42 (d, *J* = 8.2 Hz, 2H), 8.17 (t, *J* = 7.7 Hz, 2H), 8.10 (t, *J* = 8.7 Hz, 5H), 8.00 (s, 1H), 7.36 (d, *J* = 8.7 Hz, 2H), 7.25 (t, *J* = 7.4 Hz, 2H), 7.20 (t, *J* = 7.5 Hz, 2H), 6.78 – 6.71 (m, 7H), 4.99 (s, 2H), 4.49 (s, 2H), 4.06 (s, 5H). **^{13}C NMR (101 MHz, DMSO-*d*$_6$)** δ 168.17, 166.19, 159.27, 159.03, 149.58, 148.62, 147.57, 145.93, 145.82, 145.77, 143.89, 143.84, 143.79, 143.72, 139.24, 139.16, 136.23, 134.52, 133.49, 133.46, 130.79, 129.96, 128.88, 128.81, 128.06, 127.48, 127.27, 127.22, 126.67, 125.48, 124.96, 124.87, 124.01, 123.82, 123.71, 123.16, 122.41, 79.69, 73.51, 72.67, 69.98, 69.87, 40.46. **MALDI-TOF-MS:** calcd. for $C_{65}H_{41}FeIrN_5S_2^+$ 1205.181, found: 1205.542. Elemental analysis: calcd for $C_{65}H_{41}F_6FeIrN_5PS_2$: C 57.86, H, 3.06, N 5.19, found C 57.72, H 3.12, N 5.37.

Synthesis of Ir-NH_2: Ir-NH_2 was obtained by stirring Ir-Fc in an acidic buffer solution (pH = 6.5) and purified by column chromatography on silica gel. **^{1}H NMR (400 MHz, DMSO-*d*$_6$)** δ 9.42 (t, *J* = 7.9 Hz, 2H), 8.98 (d, *J* = 4.9 Hz, 1H), 8.83 (d, *J* = 7.4 Hz, 2H), 8.74 (d, *J* = 8.5 Hz, 1H), 8.48 (dd, *J* = 13.8, 6.6 Hz, 3H), 8.14 (dt, *J* = 24.0, 8.2 Hz, 8H), 7.92 (dd, *J* = 8.4, 5.3 Hz, 1H), 7.64 (dd, *J* = 8.3, 5.2 Hz, 1H), 7.34 (d, *J* = 8.6 Hz, 1H), 7.25 (q, *J* = 7.4 Hz, 6H), 6.78 – 6.63 (m, 10H). **^{13}C NMR (101 MHz, CD_2Cl_2)** δ 168.17, 168.12, 159.52, 158.90, 147.65, 146.91, 145.73, 145.62, 143.99, 143.93, 143.87, 143.77, 143.55, 142.81, 141.14, 139.14, 139.00, 135.75, 133.66, 133.50, 133.42, 133.37, 132.09, 130.93, 128.91, 128.83, 128.73, 128.10, 128.00, 127.49, 127.45, 126.91, 126.58, 126.54, 125.50, 124.83, 124.75, 124.72, 124.70, 124.28, 123.48, 122.69, 122.65, 122.61, 122.57, 122.52, 122.49, 122.22, 104.05. **MALDI-TOF-MS:** calcd. for $C_{54}H_{33}IrN_5S_2^+$ 1009.180, found: 1009.582.

NMR and MALDI-TOF-MS spectra of compounds

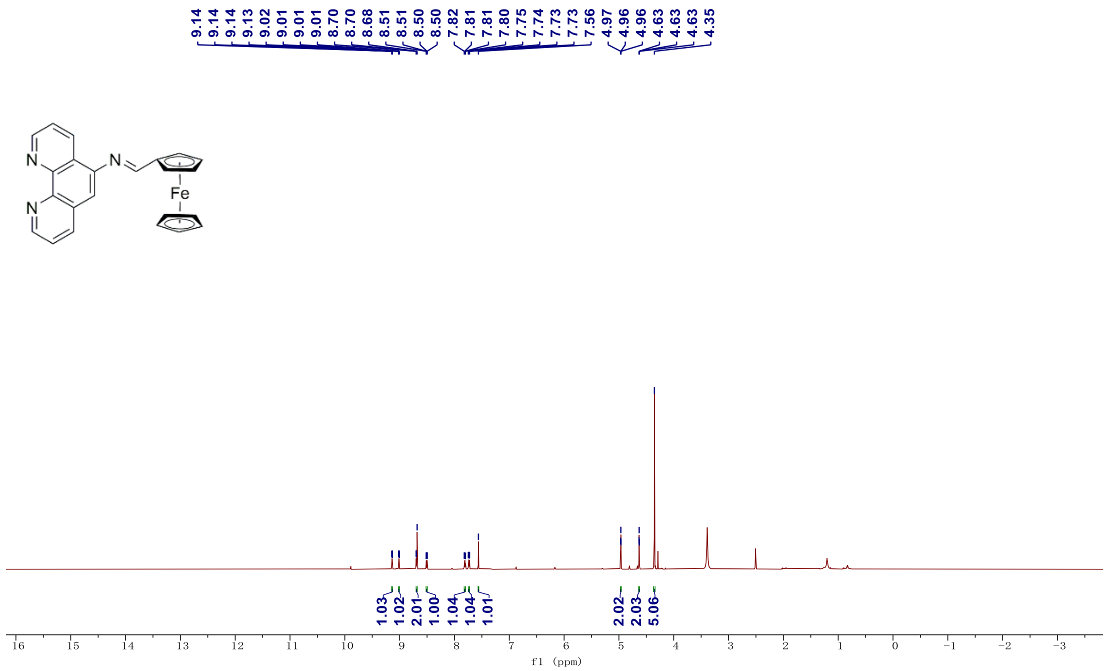

Figure S1. ^{1}H NMR spectrum of L-Fc in DMSO-*d*$_6$.

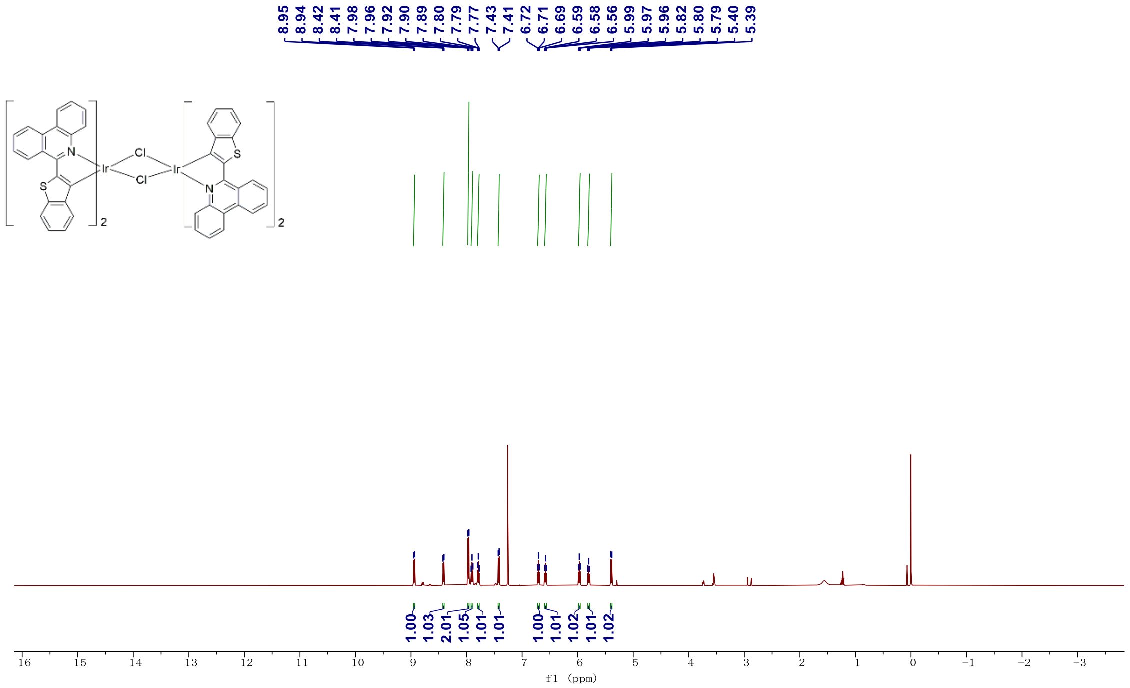

Figure S2. ^1H NMR spectrum of $Ir_2(btph)_4\mu$-Cl_2 in $CDCl_3$.

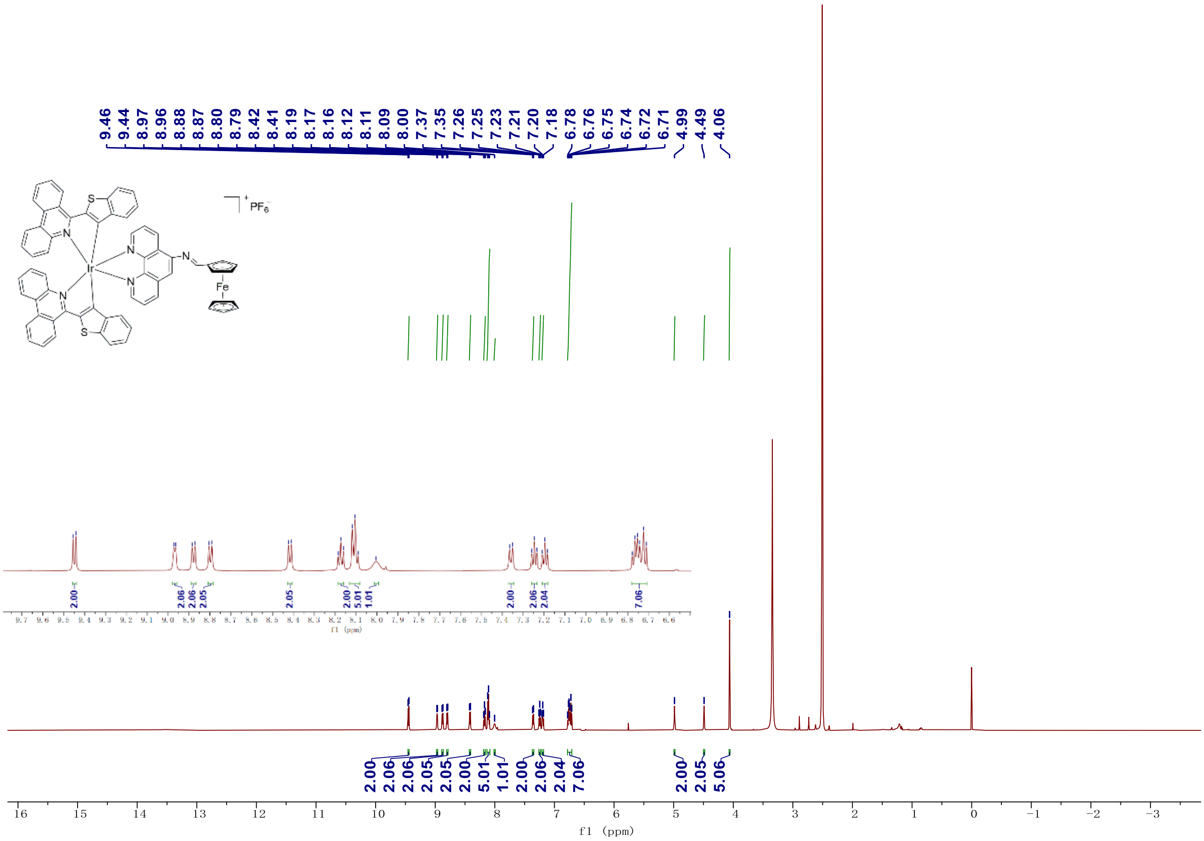

Figure S3. ^1H NMR spectrum of Ir-Fc in DMSO-d_6.

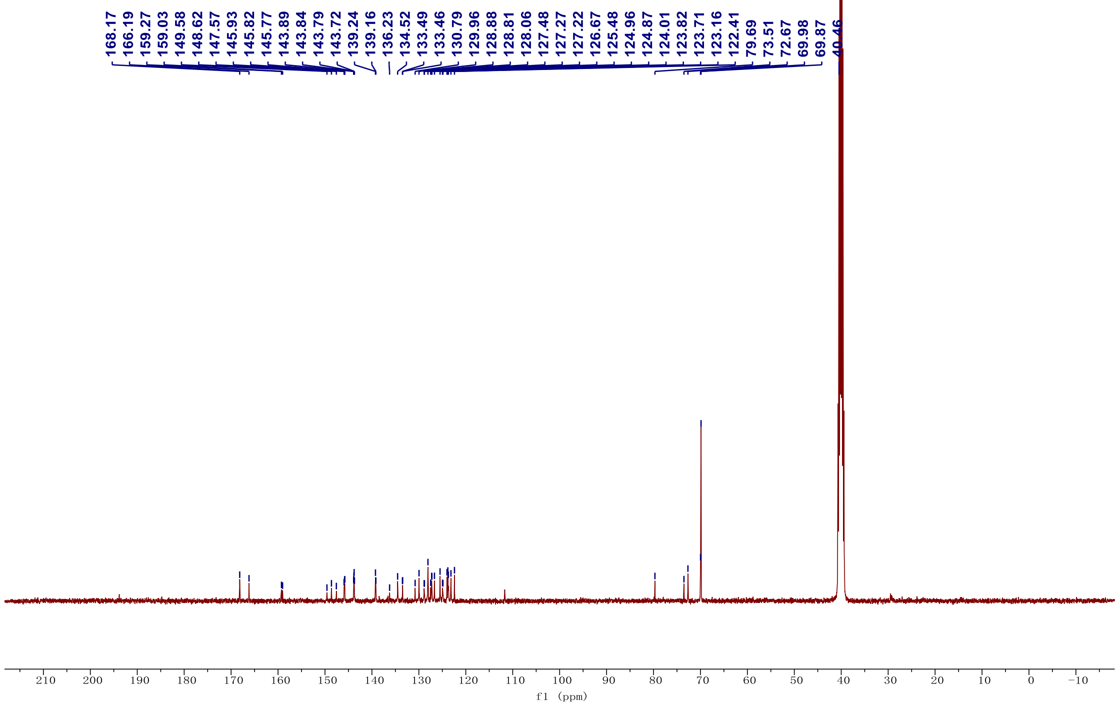

Figure S4. ^{13}C NMR spectrum of Ir-Fc in DMSO-d_6.

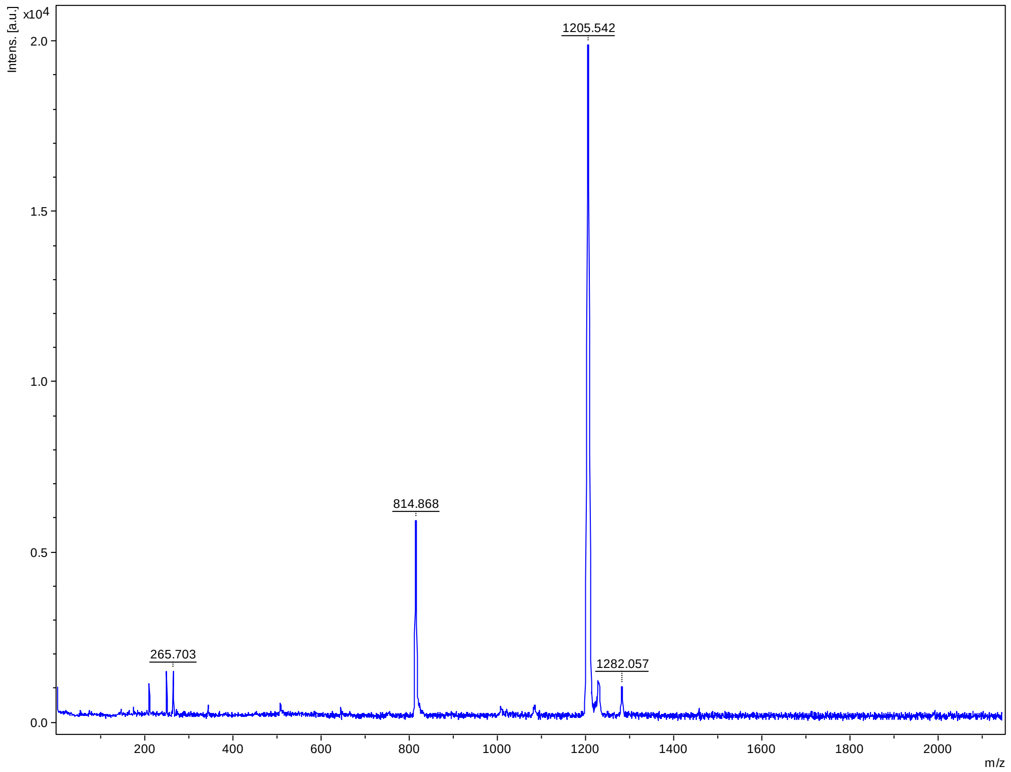

Figure S5. MALDI-TOF-MS spectrum of Ir-Fc.

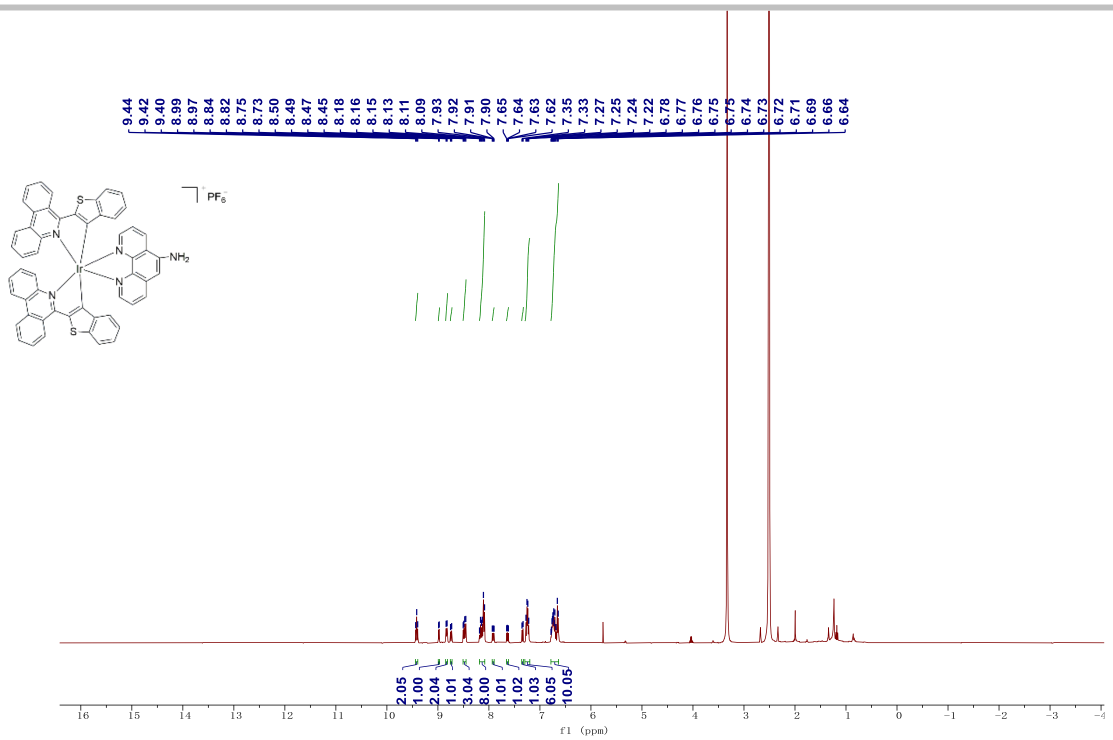

Figure S6. ^{1}H NMR spectrum of Ir-NH_2 in DMSO-d_6.

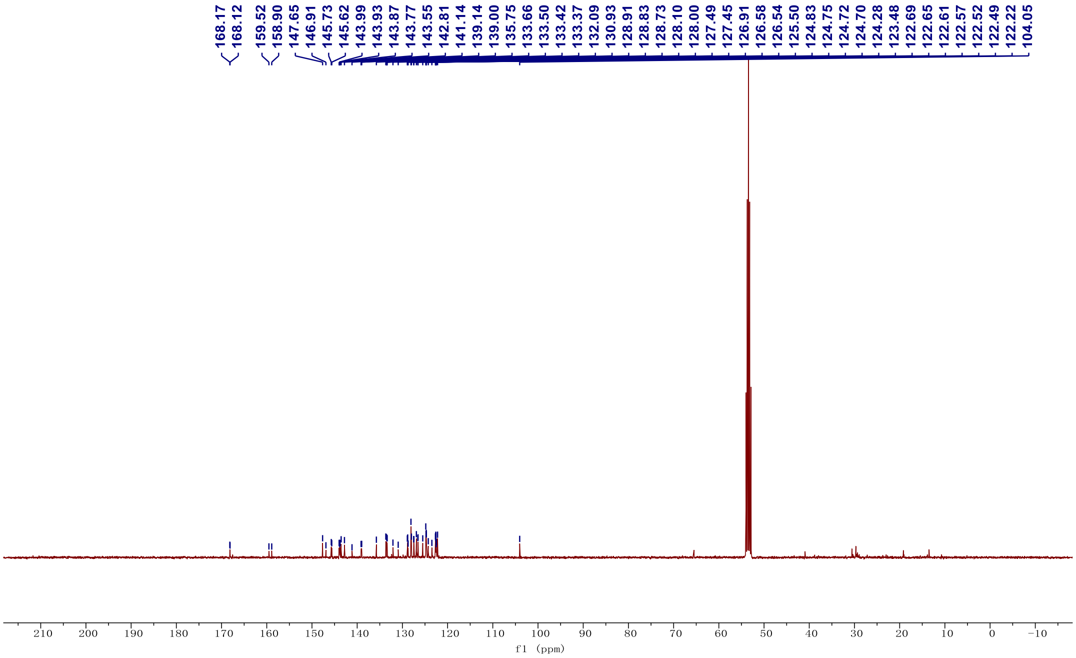

Figure S7. ^{13}C NMR spectrum of Ir-NH_2 in CD_2Cl_2.

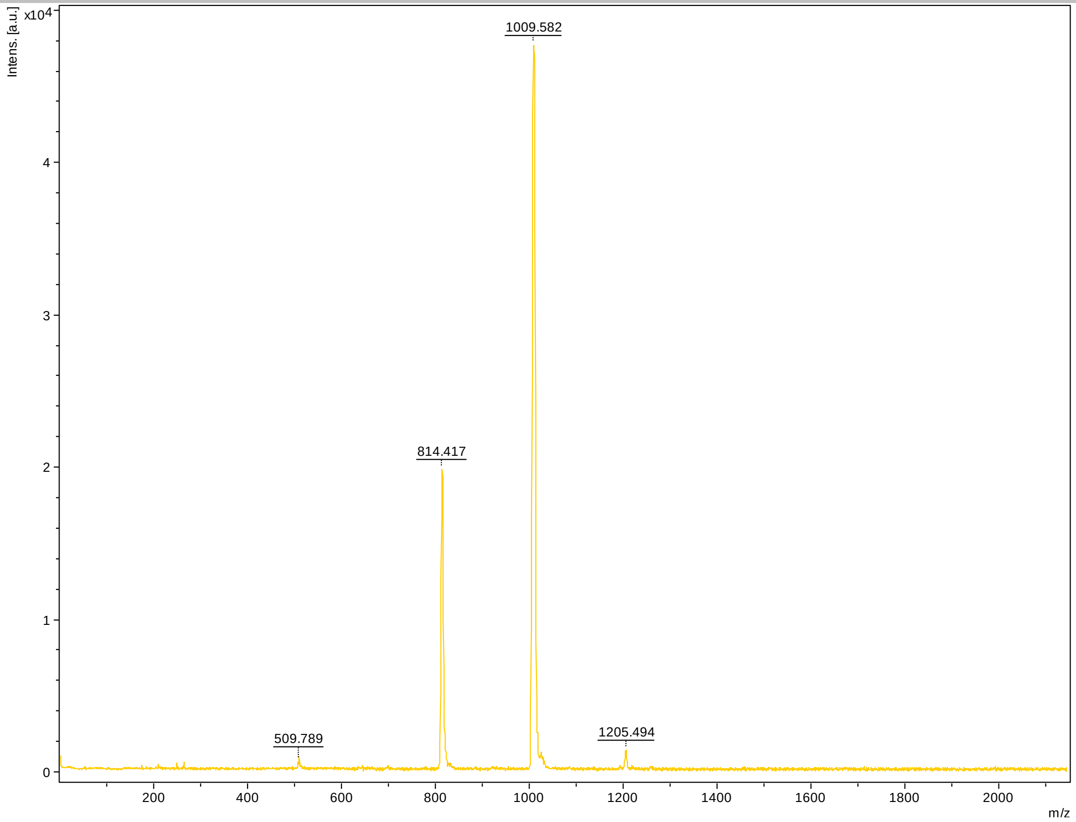

Figure S8. MALDI-TOF-MS spectrum of Ir-NH_2.

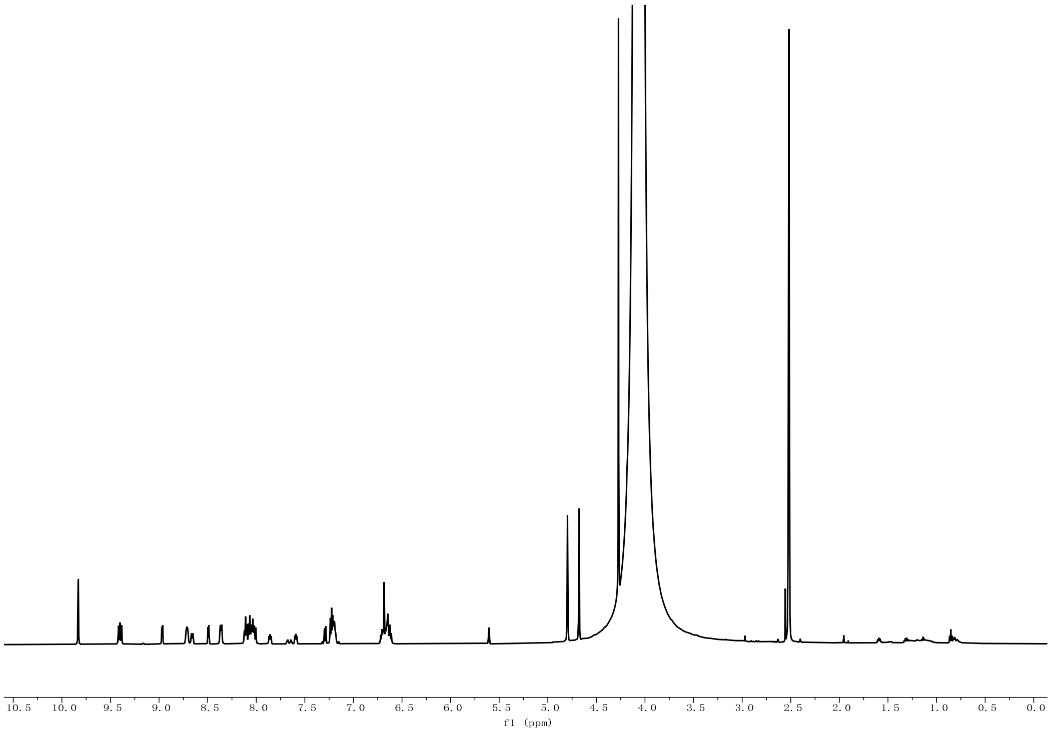

Figure S9. ^{1}H NMR spectrum of Ir-Fc + 0.01 ml HCl in DMSO-d_6.

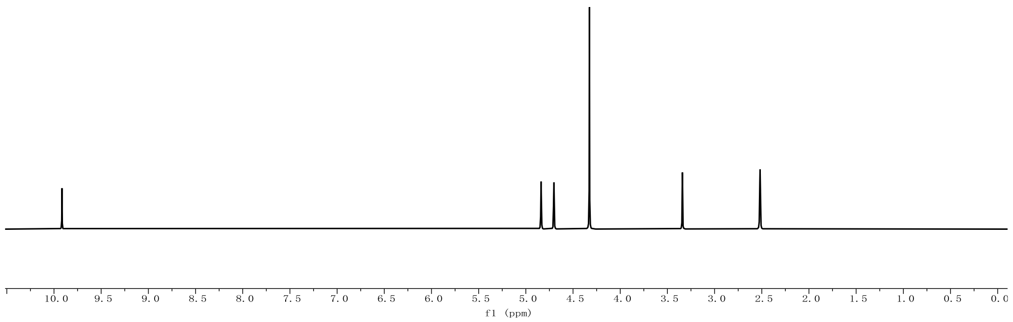

Figure S10. ^1H NMR spectrum of Fc-CHO in DMSO-d_6.

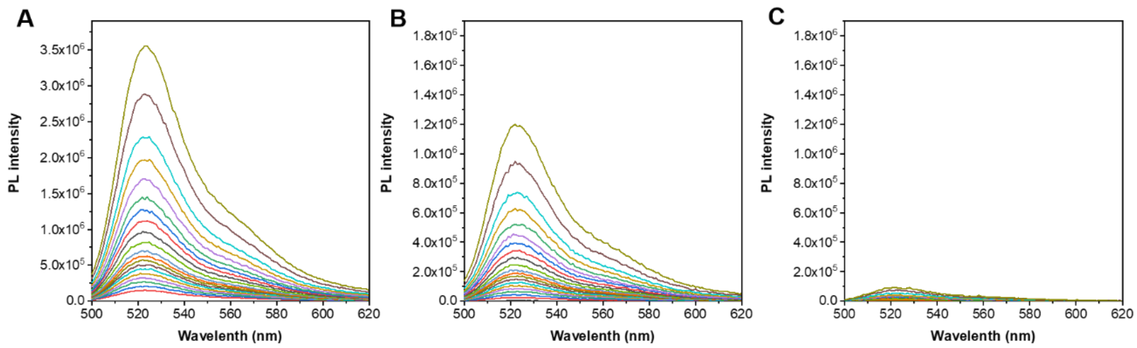

Figure S11. PL spectra of DCFH in the presence of (A) DCFH + Ir-NH_2 (0.1 μM), (B) DCFH + Ir-Fc (0.1 μM), (C) DCFH when irradiated by white LEDs (20 mW cm^{-2}).

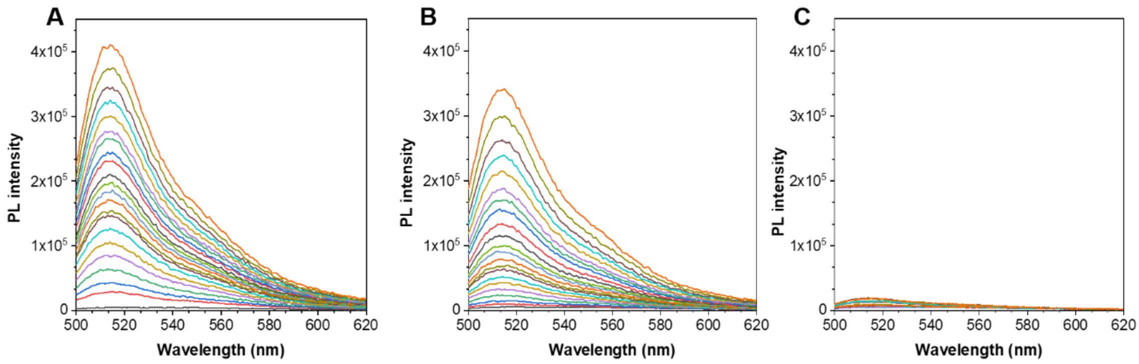

Figure S12. PL spectra of HPF in the present of (A) HPF + Ir-NH_2 (0.1 μM), (B) HPF + Ir-Fc (0.1 μM), (C) HPF when irradiated by white LEDs (20 mW cm^{-2}).

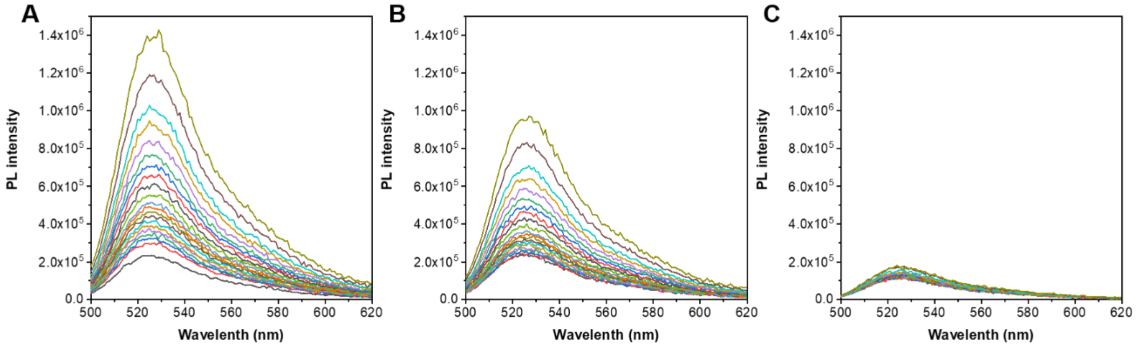

Figure S13. PL spectra of DHR 123 in the present of (A) DHR 123 + Ir-NH_2 (0.1 μM), (B) DHR 123 + Ir-Fc (0.1 μM), (C) DHR 123 when irradiated by white LEDs (20 mW cm^{-2}).

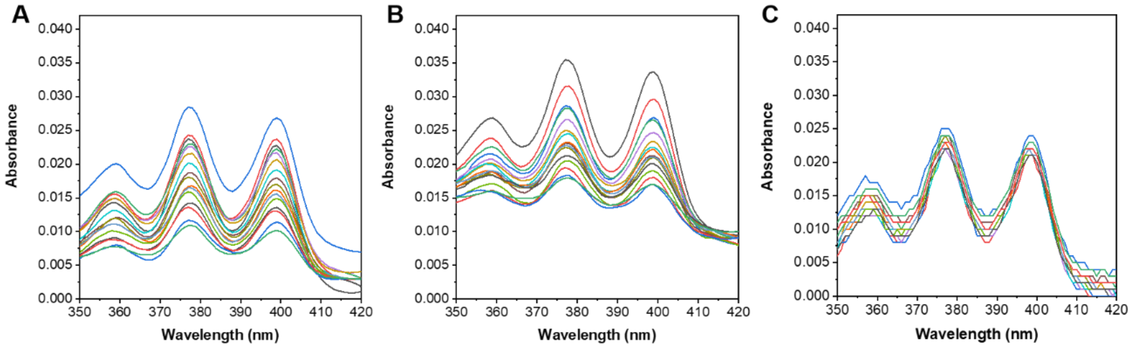

Figure S14. Absorption spectra of ABDA in the presence of (A) ABDA + Ir-NH_2 (0.1 μM), (B) ABDA + Ir-Fc (0.1 μM), (C) ABDA when irradiated by white LEDs (20 mW cm^{-2}).

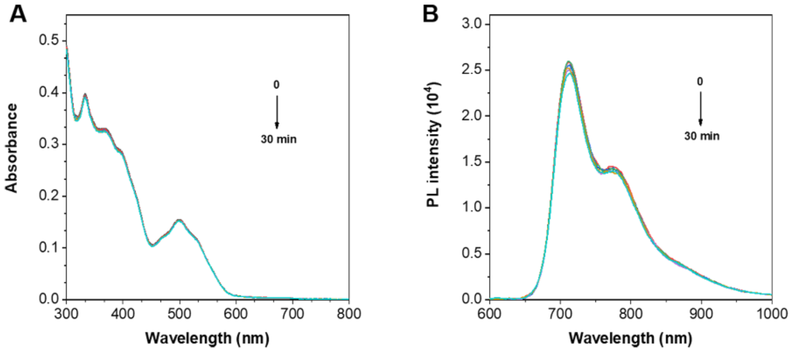

Figure S15. UV-vis absorption and PL spectral changes of Ir-NH_2 upon exposure to white LEDs (20 mW cm^{-2}) for 30 min.

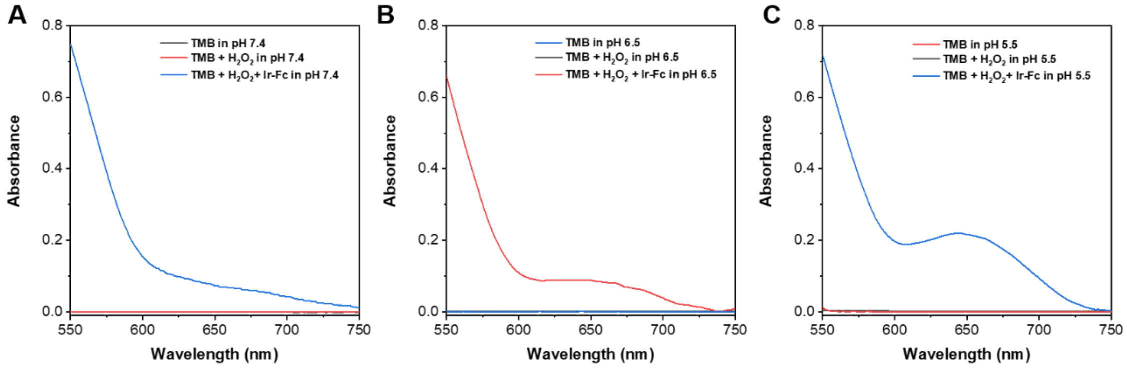

Figure S16. Absorption spectra of TMB solution treated with Ir-Fc (10 μM) and H_2O_2 (90 μM) at different pH values.

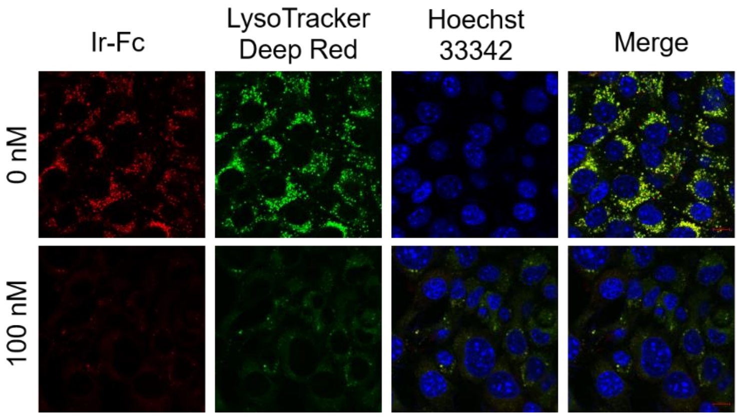

Figure S17. Colocalization images of Ir-Fc (30 μM) in the presence or absence of bafilomycin A1 (BafA1) (100 nM, 1 h) in 4T1 cells, Scale bar = 10 μm.

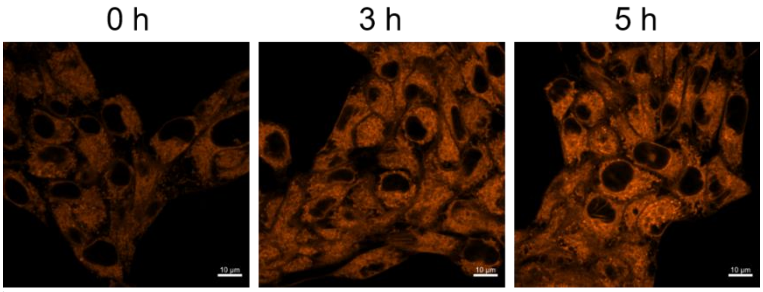

Figure S18. Intracellular Fe^{2+} production at different incubation times (red fluorescence: FerroOrange; scale bar: 10 μm).

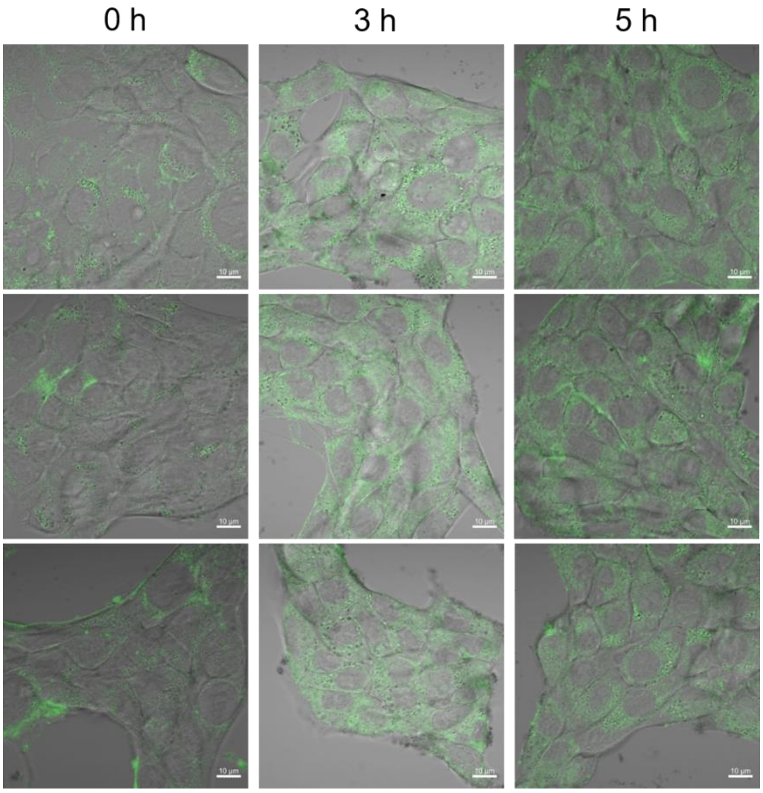

Figure S19. Confocal fluorescence images for detecting the intracellular LPO level in 4T1 cells treated with Ir-Fc (30 μM) and Liperfluo as fluorescent probe. Scale bar = 10 μm.

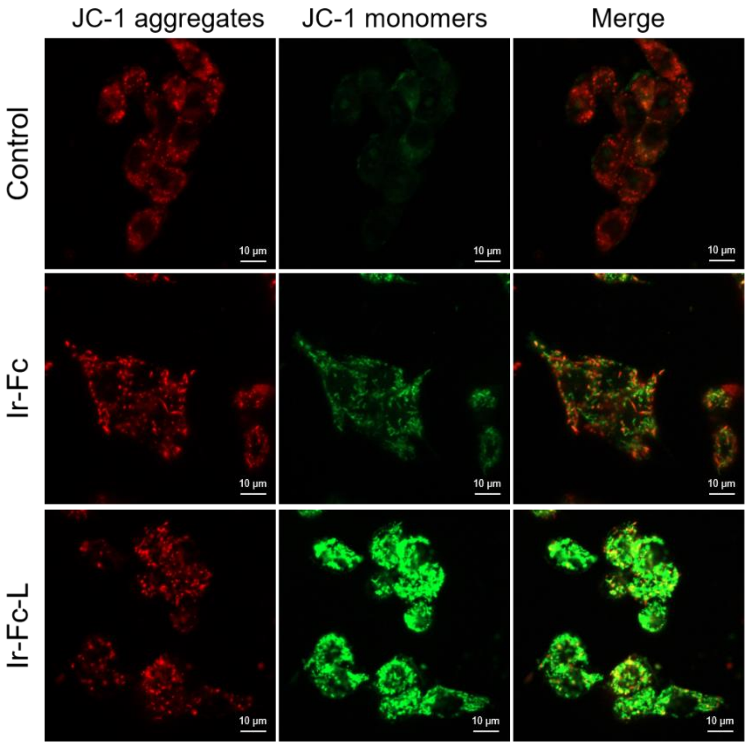

Figure S20. Confocal fluorescence imaging of MMP in 4T1 cells incubated with Ir-Fc (10 μM) and Ir-Fc + L (white LEDs 20 mW cm^{-2}) via JC-1 dye assay. Scale bar = 10 μm.

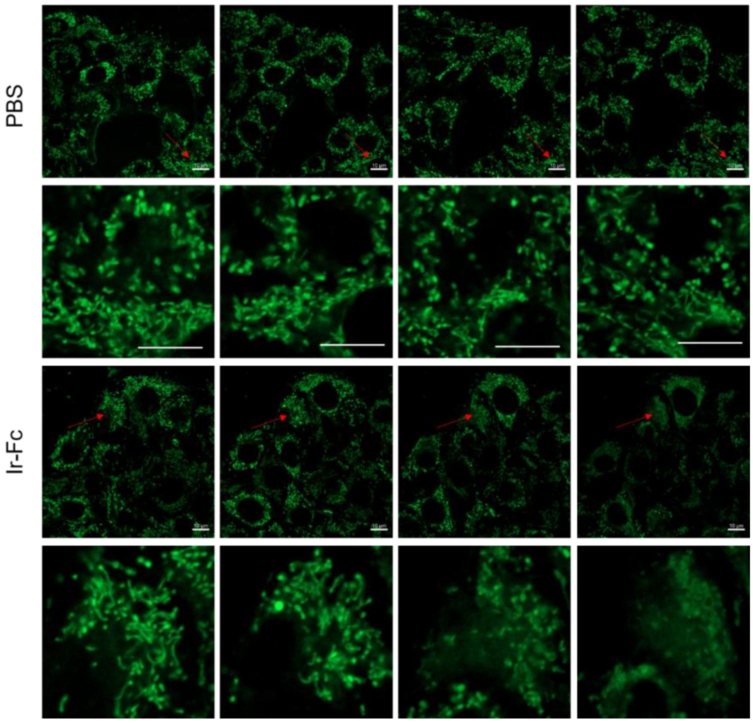

Figure S21. Real-time tracking of mitochondrial morphology in 4T1 cells treated with PBS and Ir-Fc (10 μM) for 90 min.

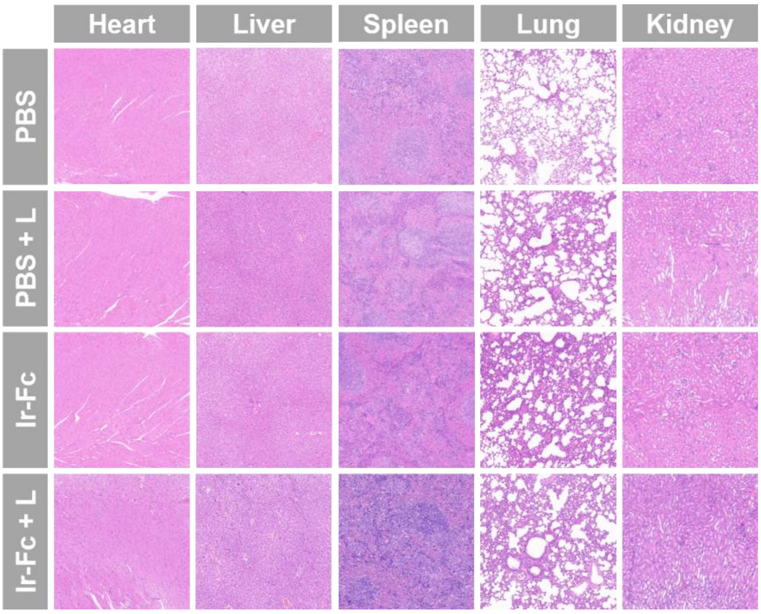

Figure S22. Histological images of the vital organs of 4T1 tumor-bearing mice on day 15 post different interventions.

Table S1. Photophysical characteristics of Ir-Fc and $Ir\text{-}NH_2$.

Complexes	$\lambda_{abs,\ max}$ (nm)	$\lambda_{em,\ max}$ (nm)	Φ_{PL}	τ (ns)	$k_r \times 10^6\ s^{-1}$	$k_{nr} \times 10^6\ s^{-1}$
Ir-Fc	500	712	0.02	295.57	0.07	0.27
$Ir\text{-}NH_2$	500	712	0.06	292.32	0.21	0.14

Measured in CH_3CN (1×10^{-5} M) solution; λ_{ex} 500 nm.

Table S2. Routine blood indexes of mice after different treatments.

	PBS	PBS+L	Ir-Fc	Ir-Fc+L	Reference range
WBC (10^9/L)	72.6±31.1	103.1±39.8	100.6±49.2	8.95±2.6	0.8-10.6
Lymph (10^9/L)	62.4±26.9	85.6±26.9	77.55±33.6	4.85±1.2	0.6-8.9
Mon (%)	3.9±1.0	3.8±0.66	5.0±1.1	6.5±1.38	0.9-18
Gran (10^9/L)	7.6±3.6	13.5±10.9	17.95±13.8	2.8±0.7	0.23-3.6
RBC (10^{12}/L)	8.6±0.7	8.63±0.66	8.27±0.45	9.90±2.39	6.5-11.5
HGB (g/L)	130.2±15.7	135.5±6.3	130.2±3.4	136.8±7.5	110-165
HCT (%)	40.1±3.6	40.48±3.3	39.3±2.0	41.32±2.8	35-55
MCV (fL)	46.43±0.5	46.93±0.8	47.6±0.8	46.68±1.0	41-55
MCH (pg)	14.9±0.7	15.7±0.8	15.7±0.5	14.3±2.5	13-18
MCHC (g/L)	323.7±14.5	335.1±18.1	330.7±9.7	332.4±2.7	300-360
RDW (%)	14.7±0.3	14.5±0.6	14.8±0.5	14.7±1.3	12-19

PLT (10^9/L)	1155.4±310.6	1165.8±243.8	1244.5±290.1	1161.6±106.1	400-1600
MPV (fL)	6.6±0.9	6.2±0.05	6.2±0.2	5.88±0.1	4.0-6.2
PDW	17.1±0.7	17.01±0.3	16.9±0.3	16.9±0.4	12.0-17.5

References

[1] a) Q. Zhang, R. Cao, H. Fei, M. Zhou, *Dalton Trans.* **2014**, *43*, 16872-16879; b) X. Zheng, X. Wang, H. Mao, W. Wu, B. Liu, X. Jiang, *Nat. Commun.* **2015**, *6*, 5834.
